# Advances in the diagnosis and management of abdominoscrotal hydrocele: a narrative review

**DOI:** 10.3389/fped.2026.1865167

**Published:** 2026-08-13

**Authors:** Fangyuan Li, Lina Zhang, Yalong Ma, Jinsong Sun, Baohua Yu

**Affiliations:** 1 Clinical Medical College of Jining Medical University (Affiliated Hospital), Jining, Shandong, China; 2 Department of Obstetrics, Gynecology and Pediatrics Operating Room, Affiliated Hospital of Jining Medical University, Jining, Shandong, China; 3 Department of Pediatric Surgery, Affiliated Hospital of Jining Medical University, Jining, Shandong, China

**Keywords:** abdominoscrotal hydrocele, pediatric surgery, processus vaginalis, surgical management, ultrasonography

## Abstract

Abdominoscrotal hydrocele (ASH) is a rare variant of hydrocele characterized by contiguous scrotal and abdominal fluid collections communicating through the inguinal canal. Its pathogenesis remains poorly understood, and the available evidence is derived primarily from case reports and small retrospective case series, leaving continued uncertainty over its diagnosis, timing of intervention, and operative strategy. This narrative review summarizes and critically discusses the current literature on the pathogenesis, clinical presentation, imaging evaluation, differential diagnosis, and management of pediatric ASH. In children presenting with an apparently simple hydrocele, an unusually large, tense, or progressively enlarging inguinoscrotal swelling, ipsilateral lower abdominal fullness, or cross-fluctuation should prompt ultrasonographic assessment extending from the scrotum through the inguinal canal. Direct demonstration of continuity between the scrotal hydrocele and an abdominal fluid collection supports the diagnosis of ASH. Magnetic resonance imaging may be useful when the proximal extent or anatomical origin of the lesion remains unclear. Proposed mechanisms include pressure-driven cephalad extension of a scrotal hydrocele and persistent patency of the processus vaginalis, although neither mechanism fully accounts for all reported cases. Observation may be appropriate in carefully selected asymptomatic infants with close follow-up, whereas progressive, symptomatic, compressive, or diagnostically uncertain lesions generally warrant surgical intervention. Inguinal, scrotal, and laparoscopic approaches are all feasible, but the available evidence does not demonstrate the superiority of any single technique. Multicenter studies with standardized diagnostic criteria and long-term outcome reporting are needed to inform risk-stratified management.

## 1 Introduction

**Figure 1:**
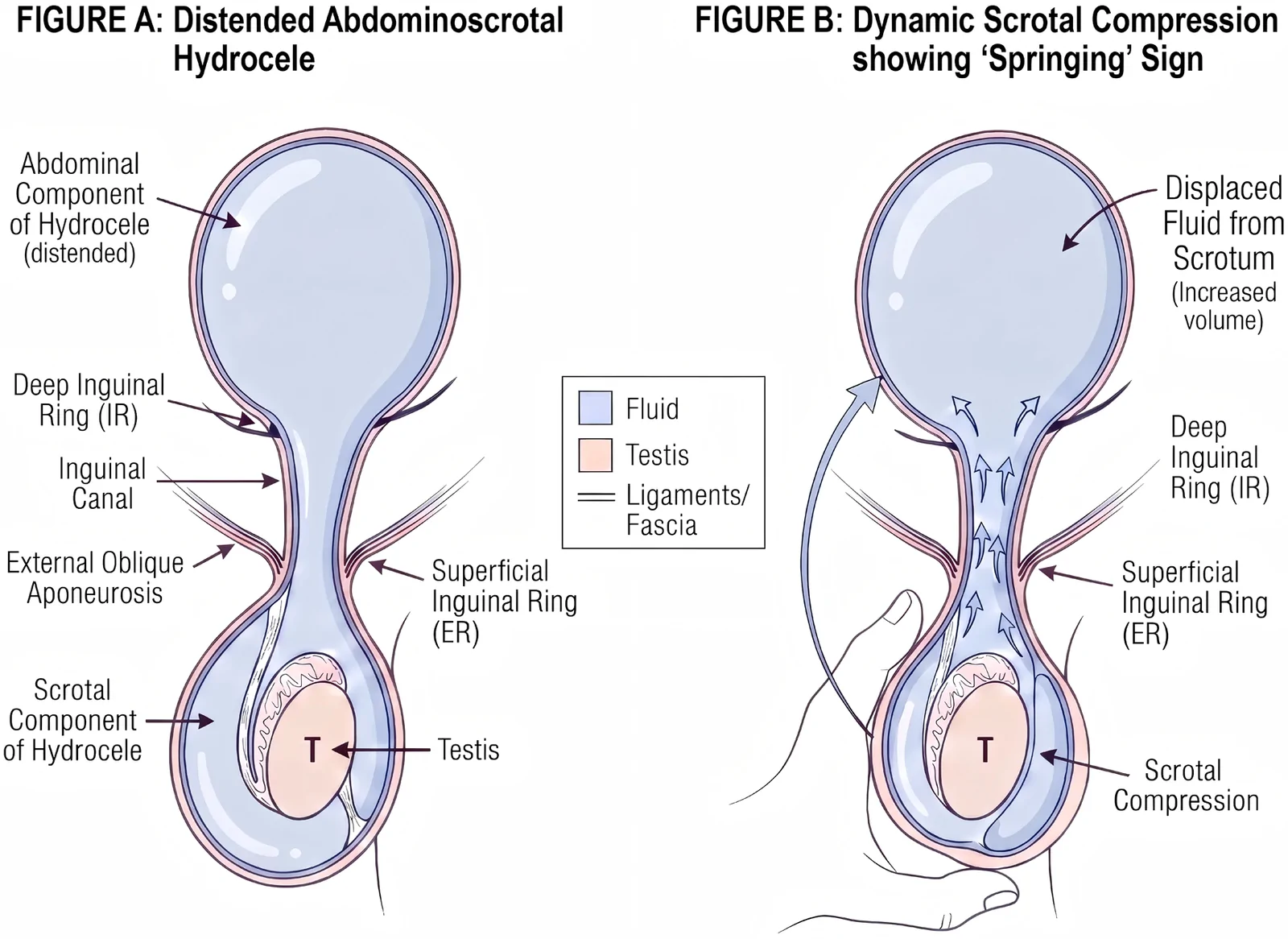
Schematic anatomy of abdominoscrotal hydrocele. **(A)** Distended abdominoscrotal hydrocele. **(B)** Dynamic scrotal compression showing “springing” sign.

Abdominoscrotal hydrocele (ASH) was first described by Dupuytren in 1834 and accounts for approximately 0.4%–3.1% of pediatric hydroceles (1, 2). ASH consists of contiguous scrotal and abdominal fluid collections connected through the inguinal canal, forming a dumbbell- or hourglass-shaped lesion (Figure 1) (1, 2). Ultrasonography is the first-line imaging modality, whereas magnetic resonance imaging may be helpful for evaluating anatomically complex or diagnostically indeterminate cases (1–3). Management strategies vary and encompass observation, open or laparoscopic surgery, and, in selected refractory cases, sclerotherapy (1, 4). This narrative review aims to summarize and critically discuss the current literature on the pathogenesis, clinical presentation, imaging evaluation, differential diagnosis, and management of pediatric ASH, with particular emphasis on unresolved issues regarding diagnosis, treatment selection, and follow-up.

## 2 Pathogenesis: evidence and unresolved questions

The pathogenesis of abdominoscrotal hydrocele (ASH) remains incompletely understood. ASH is anatomically characterized by communicating scrotal and abdominal fluid collections linked through the inguinal canal; communication with the peritoneal cavity is variable and is not required for diagnosis (1, 5–7). Accordingly, the available evidence does not support a single pathogenetic mechanism capable of accounting for all cases.

The most commonly proposed mechanism involves pressure-driven cephalad extension of a pre-existing scrotal hydrocele. In a prospective intraoperative study involving six infants with nine ASH lesions, Bayne et al. documented elevated hydrocele pressure, testicular elongation, and epididymal abnormalities, with no communication with the peritoneal cavity being identified (5). These findings are consistent with the expansion of a pressurized, proximally closed processus vaginalis through the internal inguinal ring into the preperitoneal or retroperitoneal space. However, the small cohort size limits the generalizability of these findings.

Evidence regarding the role of a patent processus vaginalis (PPV) is less consistent. Some reports have documented ASH without peritoneal communication, whereas Funatsu et al. detected a PPV laparoscopically included in all ten patients in their series (6, 7). This discrepancy may arise from heterogeneity in pathogenesis, differences in patient selection, or variability in the intraoperative assessment of processus vaginalis patency. A PPV may permit ongoing fluid influx or coexist with ASH, but current evidence does not support its role as a universal initiating factor.

Evidence for the proposed one-way valve mechanism remains largely indirect. Cross-fluctuation or the springing-back-ball sign demonstrates fluid displacement between the scrotal and abdominal components, but it does not establish unidirectional flow from the peritoneal cavity. Other theories, such as congenital peritoneal diverticula and herniation of a pre-existing extraperitoneal cyst, are based primarily on isolated reports (1, 8).

Taken together, the available evidence favors a multifactorial pathogenesis in which persistent fluid production, impaired drainage, elevated intracystic pressure, and patient-specific inguinal anatomy collectively promote progressive abdominal extension (1, 5–8). Whether PPV-related inflow defines a distinct subtype or merely an associated finding remains unresolved.

## 3 Clinical presentation and diagnostic strategy

ASH is predominantly identified in infancy or early childhood and is typically unilateral. The typical presentation consists of a painless, tense inguinoscrotal swelling, with or without a palpable ipsilateral lower abdominal mass (1, 2, 9–11). Bilateral disease is uncommon but has been reported, including giant bilateral lesions in infancy and childhood (12, 13). However, the abdominal component may be small, deep, or clinically occult, leading to its initial misdiagnosis as an uncomplicated scrotal hydrocele.

No validated clinical prediction rule or single physical finding can reliably diagnose ASH in the absence of imaging. Therefore, the practical priority is to identify features that should prompt evaluation beyond the scrotum. Suspicion should be raised when an apparently simple hydrocele is disproportionately large or tense, shows progressive enlargement during follow-up, extends proximally toward the inguinal canal, or is associated with ipsilateral inguinal or lower abdominal fullness (1, 8–10). An ill-defined proximal border of the swelling, displacement or elongation of the ipsilateral testis, or an inability to palpate the testis separately should likewise prompt further evaluation.

When both the scrotal and abdominal components are clinically accessible, compression of either component may cause enlargement of the other, resulting in cross-fluctuation (1, 2, 10, 11). This finding supports the presence of fluid communication between the two components but is not invariably elicited. A negative cross-fluctuation test does not exclude ASH, particularly when the abdominal component is small, deeply located, tense, or partially decompressed. Accordingly, the absence of a palpable abdominal mass or cross-fluctuation should not be used to rule out ASH in patients with a large or atypical inguinoscrotal hydrocele.

Clinical complications of ASH arise primarily from persistently elevated intracystic pressure, compression of adjacent structures, or alterations in the hydrocele contents. Large or longstanding lesions may elongate or distort the ipsilateral testis and compress or displace the ureter, bladder, pelvic vessels, or lymphatic structures (1, 5, 14–16). Reported sequelae include hydroureteronephrosis, urinary tract obstruction, and lower-extremity edema or cyanosis. However, these events have been documented primarily in isolated cases or small case series, and their incidence and long-term effects on testicular growth and function remain poorly defined.

Intralesional hemorrhage is a rare yet clinically significant complication. Estevão-Costa et al. reported a child presenting with a recurrent tense inguinoscrotal hydrocele, abdominal pain, vomiting, tachycardia, pallor, and severe anemia (17). Ultrasonography and computed tomography revealed a large heterogeneous cystic mass with septations and apparent solid components; surgical exploration identified hemorrhagic fluid and blood clots within an ASH, with no evidence of malignancy. Hemorrhage may therefore lead to acute enlargement, increased tension, anemia, and heterogeneous features mimicking a complex cystic or neoplastic lesion. Accordingly, ASH should be considered in the differential diagnosis of a tense or recurrent hydrocele associated with an abdominal mass, even when the internal contents are not uniformly anechoic. These complications should therefore be regarded as potential but uncommon sequelae rather than characteristic features of ASH.

Ultrasonography is the first-line imaging modality (1–3, 9–11). For a typical small hydrocele confined to the scrotum, routine abdominal imaging is generally unnecessary. When suspicious clinical features are present, however, ultrasonography should not be confined to the scrotum. The examiner should trace the fluid collection cranially from the scrotal sac along the spermatic cord, through the inguinal canal, and into the ipsilateral lower abdomen or preperitoneal space.

A comprehensive ultrasonographic examination should document the continuity between the scrotal and abdominal components, the dimensions and proximal extent of the lesion, locularity, internal septations or debris, and lesion's relationship with adjacent structures (2, 3, 10, 11). Gentle dynamic compression of the scrotal or abdominal component may reveal fluid displacement between the two compartments. Testicular position, morphology, volume, and Doppler perfusion should also be assessed, as prolonged pressure may be associated with testicular distortion. In patients with a large abdominal component or suspected urinary tract obstruction, the ultrasonographic examination should also include the kidneys, ureters, and bladder.

The key imaging finding supporting the diagnosis of ASH is direct continuity of a fluid-filled lesion extending from the scrotum through the inguinal canal into the abdominal or preperitoneal space (1, 2, 9–11). Hydrocele size alone is not diagnostic. The clinically relevant distinction is whether an apparently isolated scrotal collection has a proximal component that extends beyond the inguinal canal.

Magnetic resonance imaging may be useful when ultrasonography cannot adequately delineate the proximal extent or anatomical origin of the lesion, or when exclusion of a complex abdominal, pelvic, or retroperitoneal cystic mass is necessary (3, 9–11). Magnetic resonance imaging may also clarify the relationship of a large lesion to the urinary tract, bowel, and major vessels. Computed tomography can provide detailed anatomical information but exposes patients to ionizing radiation and should not be routinely used in children when ultrasonography or magnetic resonance imaging provides adequate diagnostic information.

The principal differential diagnoses include communicating hydrocele, indirect inguinal hernia, spermatic cord hydrocele, lymphatic malformation, mesenteric or retroperitoneal cyst, enteric duplication cyst, bladder diverticulum, and hydronephrosis (3, 9–11, 18–20). Communicating hydroceles and indirect inguinal hernias may fluctuate in size and extend into the inguinal canal, but they do not necessarily contain a distinct abdominal cystic component continuous with the scrotal collection. A spermatic cord hydrocele is located along the cord and is usually separate from the tunica vaginalis. Lymphatic malformations are commonly multiloculated and may display infiltrative margins rather than a defined hourglass configuration; rare abdominoscrotal lymphangiomas may nevertheless mimic communicating hydroceles (19). Multiloculation alone does not rule out ASH, but extensive trans-spatial infiltration, irregular solid components, internal vascularity, or the absence of a demonstrable connection between the abdominal and scrotal components should raise suspicion for an alternative diagnosis. Thus, preoperative recognition of ASH relies not on routine advanced imaging of every hydrocele, but on identifying atypical clinical features and extending ultrasonographic assessment beyond the scrotum when indicated. Histopathological examination is not routinely required because ASH is primarily diagnosed through clinical examination and imaging. It should be reserved for atypical lesions with solid components, infiltrative features, or an abnormal cyst wall.

## 4 Management: from routine excision to individualized decision-making

### 4.1 Observation versus surgery

The natural history of infantile ASH is not yet fully characterized. Historically, early surgery was advocated because spontaneous resolution was considered uncommon and persistent pressure was associated with testicular dysmorphism and compression of adjacent structures (1, 5, 14, 21, 22). However, the available evidence does not support immediate surgical intervention as mandatory for all patients.

A recent retrospective comparative study documented spontaneous resolution in approximately one-quarter of infants managed nonoperatively (21). Earlier reports also documented partial or complete regression during observation in selected infants (22, 23). These findings suggest that observation may be a reasonable option in carefully selected asymptomatic infants with preserved testicular morphology and perfusion, no urinary tract compression, and reliable access to serial follow-up. Nevertheless, prolonged high-pressure lesions may cause progressive testicular distortion and render subsequent dissection more difficult.

Surgical intervention should be considered in the presence of progressive enlargement, pain, testicular dysmorphism or impaired perfusion, hydronephrosis or urinary tract compression, diagnostic uncertainty, an associated hernia, or an inability to ensure close follow-up (1, 5, 14, 21, 24). Because validated predictors of spontaneous resolution are lacking, management should be individualized rather than determined solely by the patient's age or lesion size.

### 4.2 Operative principles and approach selection

The principal objectives of surgery are decompression of the hydrocele, preservation of the testis, epididymis, vas deferens, and testicular vessels, management of any patent processus vaginalis (PPV) or associated hernia, and prevention of hydrocele persistence or recurrence (1, 25–29). Complete excision of both components is not necessarily required to accomplish these goals.

The inguinal approach provides direct access to the spermatic cord, internal ring, and proximal component and has traditionally been the most commonly used approach (2, 25). Its principal drawback is the difficulty of safely separating a thick or adherent hydrocele wall from the vas deferens and testicular vessels. Controlled aspiration or partial opening of a tense sac may facilitate the identification and protection of these structures.

A scrotal approach provides direct access to the distal hydrocele and may limit the extent of inguinal dissection (12, 26, 27, 30). However, management of a large proximal component or an associated PPV may be more challenging. Recent trans-scrotal and video-assisted techniques offer potential means of limiting extensive inguinal dissection in selected giant lesions, but the supporting evidence remains confined to small reports (12, 30). A combined approach may be necessary for exceptionally large, recurrent, or anatomically complex lesions, although it requires more extensive surgical exposure (28).

**Figure 2:**
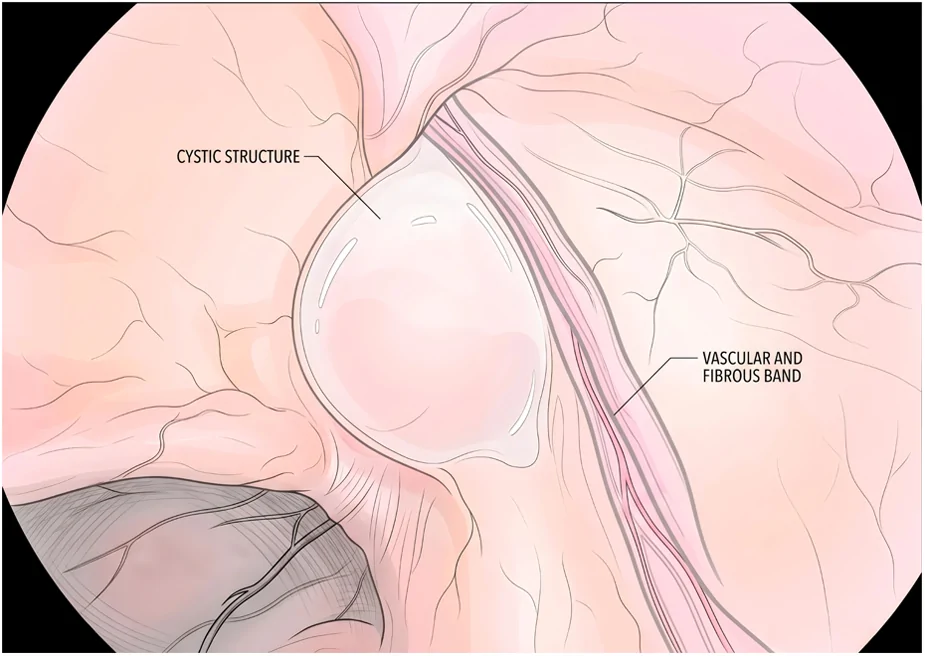
Schematic representation of the intraoperative anatomy of abdominoscrotal hydrocele. A smooth, tense, translucent cystic swelling is seen bulging into the abdominal cavity in the inguinal region.

Laparoscopy allows direct inspection of the internal rings (Figure 2), identification of contralateral abnormalities, and assessment of the relationship between the abdominal component and the peritoneal cavity (7, 28, 31–34). Diagnostic laparoscopy has also been used to delineate bilateral and noncommunicating hydrocele anatomy when preoperative findings are atypical (34). Reported laparoscopic techniques include closure of the PPV, internal ring repair, fenestration of the abdominal component, and laparoscopic-assisted excision. In a series of ten children, Funatsu et al. treated all patients using laparoscopic percutaneous extraperitoneal closure, with no recurrence observed over a mean follow-up of 2.6 years (7). These findings demonstrate the feasibility of this approach but do not establish its superiority given the small sample size and absence of a comparison group.

Accordingly, the operative approach should be individualized on the basis of the size and location of the abdominal component, the presence of a PPV or hernia, a history of previous surgery, anatomical complexity, and the surgeon's expertise (1, 7, 25–29, 31–33). Current evidence is insufficient to identify any inguinal, scrotal, combined, or laparoscopic approach as universally preferred.

### 4.3 Extent of sac treatment and salvage strategies

Complete sac excision was historically undertaken to minimize recurrence. However, extensive dissection may be unnecessary and may heighten operative risk when the sac is densely adherent to the spermatic cord or testis. Several reports have described successful outcomes following partial excision, wide fenestration, drainage, or closure of the PPV without complete removal of the sac (7, 25–29). These findings suggest that eliminating a persistent source of fluid and achieving adequate decompression may be more important than complete cyst-wall excision in selected patients.

The optimal management of the internal ring also remains uncertain. When a PPV is identified, appropriate treatment options include high ligation or laparoscopic closure (7, 24, 28, 31). Repair of a markedly enlarged internal ring may be warranted when there is concern about a subsequent inguinal hernia, but routine reinforcement is not supported by comparative evidence in pediatric patients.

Sclerotherapy has been reported as a treatment for recurrent or surgically complex ASH, including its use in a child with persistent disease after multiple operations (4). A systematic review has compared aspiration and sclerotherapy with hydrocelectomy for conventional hydroceles (35), but this evidence is not ASH-specific and cannot be directly extrapolated to pediatric ASH. The available ASH-specific evidence remains limited to isolated case reports. Sclerotherapy should therefore be considered as a salvage or investigational option rather than a routine first-line treatment.

The potential advantages, principal limitations, appropriate clinical contexts, and current evidence levels of the available management strategies are summarized in Table 1.

**Table 1:** Comparison of management strategies for abdominoscrotal hydrocele.

Strategy	Potential advantages	Main limitations	Appropriate context	Evidence level
Observation	Avoids anesthesia and surgery; possible spontaneous resolution	Requires reliable surveillance; risk of progression and delayed difficult surgery	Asymptomatic infant without compression or testicular compromise	Retrospective comparative evidence
Inguinal approach	Direct access to cord, PPV and internal ring	Difficult dissection when sac is adherent	PPV, hernia or dominant inguinal/proximal disease	Small series
Scrotal approach	Direct distal access; limited inguinal dissection	Limited control of high abdominal component or PPV	Predominantly scrotal lesions	Small series
Laparoscopic approach	Visualizes internal rings and contralateral pathology	Technical variation; no proven superiority	PPV, uncertain anatomy or bilateral assessment	Small series
Combined approach	Broad exposure for complex lesions	Greater operative trauma	Giant, recurrent or anatomically complex ASH	Case reports/series
Sclerotherapy	Avoids extensive repeat dissection	Unknown pediatric safety and recurrence profile	Refractory or high-risk cases	Isolated cases

ASH, abdominoscrotal hydrocele; PPV, patent processus vaginalis. The evidence levels shown in this table reflect the predominant study designs available in the published literature rather than a formal evidence-grading system. Selection of the management strategy should be individualized according to the patient's age, symptoms, lesion size and extent, testicular or urinary tract involvement, presence of a PPV or associated inguinal hernia, previous treatment, and the surgeon's experience.

### 4.4 Perioperative complications and follow-up

The risk of surgical morbidity varies according to lesion size, prior surgery, and the degree of adherence between the hydrocele wall and the vas deferens, epididymis, or testicular vessels (1, 25, 28, 29). Extensive dissection performed to achieve complete sac excision may increase the risk of vascular or ductal injury, although the true incidence of these events remains unknown because most published studies are small and retrospective.

Reported early postoperative complications include scrotal or inguinal edema, hematoma, postoperative pain, and wound-related morbidity. One small series of eight patients documented two postoperative hematomas and one recurrent hydrocele (28). Other series have described only transient scrotal edema or no clinically significant early complications (27, 29), but these observations should be interpreted cautiously because of limited sample sizes and inconsistent outcome reporting.

Potential late complications include hydrocele persistence or recurrence, testicular atrophy or reduced testicular volume, acquired testicular ascent, ipsilateral inguinal hernia, and unplanned reoperation (21, 28, 29). Injury to the vas deferens or testicular vessels is an additional concern when the sac is densely adherent or when extensive inguinal or abdominal dissection is required. However, most studies have not evaluated these complications using standardized definitions or sufficiently prolonged follow-up.

A multicenter retrospective study of 61 boys documented postoperative complications in 18% of patients treated with sac excision or ligation, whereas no complications were reported among those treated with limited scrotal excision and eversion (29). The difference was not statistically significant, and neither group developed a recurrent hydrocele. These findings support the feasibility of limited sac treatment and suggest that complete excision may not be required in every patient, but they do not establish the superiority of either technique.

Postoperative follow-up should include assessment of residual or recurrent inguinoscrotal swelling, testicular position, testicular volume and morphology, Doppler perfusion, the development of an ipsilateral inguinal hernia, and the need for additional intervention (21, 27–29). Longer follow-up is particularly important in patients with preoperative testicular distortion, recurrent ASH, previous inguinal surgery, or technically difficult dissection.

Because perioperative complications and long-term outcomes have been inconsistently reported, reliable comparisons among inguinal, scrotal, combined, and laparoscopic approaches cannot currently be made (1, 21, 29). Future studies should use standardized definitions of recurrence, testicular atrophy, testicular ascent, vascular or vasal injury, inguinal hernia, and reoperation, and should include objective assessment of postoperative testicular growth.

## 5 Evidence limitations and future directions

Interpretation of the ASH literature is constrained by the rarity of the condition, inconsistent terminology, and substantial heterogeneity in diagnostic and treatment approaches (1, 21, 29). Most publications consist of individual case reports or small retrospective case series, and the reported absence of recurrence may reflect short follow-up, selective reporting, or limited statistical power rather than genuine equivalence among techniques.

Several clinically important questions remain unresolved. Reliable predictors of spontaneous resolution have not been identified, and the effects of lesion duration and pressure on testicular growth remain poorly quantified (1, 5, 14, 21, 29). The relationship between ASH and PPV also remains unclear because both communicating and apparently noncommunicating lesions have been reported (6, 7). In addition, studies use inconsistent definitions of complete excision, partial excision, fenestration, and internal ring repair, hindering meaningful comparisons among operative strategies.

Future multicenter studies should adopt standardized diagnostic criteria and systematically document lesion size, locularity, PPV status, internal ring diameter, testicular volume, testicular morphology, and Doppler perfusion (1, 21, 29). Outcomes should include recurrence, reoperation, testicular growth, postoperative atrophy, hydrocele persistence, hernia formation, and length of follow-up. A prospective registry may be more feasible than a randomized trial and could provide more robust evidence to inform risk-stratified observation and operative approach selection.

## 6 Conclusion

ASH is a rare anatomical variant characterized by connected abdominal and inguinoscrotal hydrocele components. Dynamic ultrasonography is usually sufficient to establish the diagnosis and should also include assessment of the testis and adjacent urinary tract. The pathogenesis is likely heterogeneous, and neither PPV nor a one-way valve mechanism can account for all cases.

Management should be individualized. Observation may be considered for carefully selected asymptomatic infants, whereas progressive, symptomatic, compressive, or diagnostically uncertain lesions generally warrant surgery. Inguinal, scrotal, and laparoscopic approaches are all feasible, but no technique has been demonstrated as superior. Preservation of the testis and spermatic cord structures, rather than complete sac excision, should remain the principal operative priority.
